# Isolation of a Novel Low-Temperature-Active and Organic-Solvent-Stable Mannanase from the Intestinal Metagenome of *Hermetia illucens*

**DOI:** 10.3390/ijms26010216

**Published:** 2024-12-30

**Authors:** Dong-Gwan Kim, Chang-Muk Lee, Young-Seok Lee, Sang-Hong Yoon, Su-Yeon Kim

**Affiliations:** 1 Department of Bioindustry and Bioresource Engineering, Sejong University, Seoul 05006, Republic of Korea; 2 Plant Engineering Research Institute, Sejong University, Seoul 05006, Republic of Korea; 3 Technology Services Division, National Institute of Agricultural Sciences, Rural Development Administration, Wanju 55365, Republic of Korea; 4 Metabolic Engineering Division, National Institute of Agricultural Sciences, Rural Development Administration, Wanju 55365, Republic of Korea; leeys11@korea.kr (Y.-S.L.); shyo556@naver.com (S.-H.Y.); suyeon4617@gmail.com (S.-Y.K.)

**Keywords:** mannanase, metagenome, low-temperature active, organic-solvent stable

## Abstract

The black soldier fly, *Hermetia illucens*, is a voracious scavenger of various organic materials; therefore, it could be exploited as a biological system for processing daily food waste. In order to survey novel hydrolytic enzymes, we constructed a fosmid metagenome library using unculturable intestinal microorganisms from *H. illucens*. Through functional screening of the library on carboxymethyl cellulose plates, we identified a fosmid clone, the product of which displayed hydrolytic activity. Sequence analysis of the fosmid revealed a novel mannan-degrading gene, *ManEM6*, composed of 1185 base pairs encoding 394 amino acids, with a deduced 20-amino-acid *N*-terminal signal peptide sequence. The conceptual translation of *ManEM6* exhibited the highest identity (78%) to endo-1,4-β-mannosidase from *Dysgonomonas mossii*. Phylogenetic and domain analyses indicated that *ManEM6* encodes a novel mannanase with a glycoside hydrolase family 26 domain. The recombinant protein rManEM6 showed its highest activity at 40 °C and pH 7.0, and it remained stable in the range of pH 5–10.0. rManEM6 hydrolyzed substrates with β-1,4-glycosidic mannoses, showing maximum enzymatic activity toward locust bean gum galactomannan, while it did not hydrolyze *p*-nitrophenyl-β-pyranosides, demonstrating endo-form mannosidase activity. rManEM6 was highly stable under stringent conditions, including those of polar organic solvents, as well as reducing and denaturing reagents. Therefore, ManEM6 may be an attractive candidate for the degradation of mannan under high-organic-solvent and protein-denaturing processes in the food and feed industries.

## 1. Introduction

The polyphagous insect *Hermetia illucens*, also known as the black soldier fly (BSF), has excellent digestive ability; in particular, its larvae can convert food waste and manure into biomass in a few hours. With the intent of solving environmental issues, such as soil contamination and water pollution, BSF larvae-mediated bioremediation has drawn worldwide attention as an economical and sustainable method [1,2]. Despite the enormous industrial potential of BSF larvae, the biochemical and molecular potential of microbial waste-hydrolyzing enzymes in the larval gut are not well understood because many gut microorganisms are unculturable. Recently, metagenomic approaches have provided tools for surveying novel genes encoding unique biocatalysts, without specific cultivation of gut microbes [3,4,5,6,7], from mine drainage metagenome sources [8].

More than 90% of plant biomass is composed of cellulose, hemicellulose, and lignin, in an approximate ratio of 2:1:1. Second to cellulose, hemicellulose is an important source of sugars in biotechnological applications. The two most abundant and typical hemicelluloses are β-1,4-d-xylans and β-1,4-d-mannans; while xylans represent the predominant hemicellulosic component in hardwood, mannans are most abundant in softwoods and plant seeds [9]. β-1,4-d-mannan polysaccharides include pure mannans (mainly β-1,4-linked mannose) and glucomannans, which are heterogeneous conjugates of β-1,4-bound mannose, plus β-1,4-d-glucose units. When α-1,6-linked galactose side chains were added to mannan, they were classified as galactomannan [10].

Mannans and heteromannans serve as both structural components and carbohydrate reserves in plants. Hydrolyzed mannans obtained from mannan-rich plant hemicelluloses have been widely used in biotechnological applications; for instance, some of these fermentable sugars are well-known as thickening, stabilizing, and gelling agents in the food industry [11]. Moreover, hydrolyzed mannans, such as manno-oligosaccharides, provide significant multi-faceted prebiotics, which has health-promoting effects on both humans and livestock animals [12,13]; however, due to the absence of required enzymes, mammals cannot readily digest heterogeneous mannans in the small intestine [14].

Mannan-degrading mannanases play an important role in the enzymatic hydrolysis of plant polysaccharides in various areas of the pharmaceutical, food, feed, textile, and oil industries [15]. Low-temperature-active mannanases are of particular potential value for environmentally friendly and economically efficient industrial fields, where cold temperatures are required to prevent energy loss, microbial contamination, and alteration/deterioration of product quality. In this regard, β-mannanases exhibiting enhanced robustness are of great interest, as they are more able to withstand harsh industrial processes [16].

There are three major enzymes capable of degrading mannans: β-mannanase (H59, mannan endo-β-1,4-mannosidase, EC 3.2.1.78); β-mannosidase (H19, β-1,4-mannosidase, EC 3.2.1.25); and β-glucosidase (H15, β-glucosidase, EC 3.2.1.21) [15]. Mannan-degrading enzymes are further classified as either exo- or endo-type enzymes; among them, β-mannanases have so far been classified in glycoside hydrolase (GH) families 5, 26, 113, and 134 (carbohydrate-active enzyme (CAZy) database; http://www.cazy.org, accessed on 4 November 2024) [17].

After its initial categorization [18], a total of 902 β-mannanase (EC 3.2.1.78) enzyme class sequences have been deposited into the UniProt Knowledge Base with unique accession numbers (http://www.uniprot.org, accessed on 4 November 2024); however, only 60 sequences were reviewed and curated by the Swiss-Prot database, and most of the sequences (842 among 902 sequences) have not yet been fully reviewed; moreover, only 151 β-mannanase sequences have been subjected to biochemical, physicochemical, or biological functional analyses (http://www.brenda-enzymes.org/enzyme.php?ecno=3.2.1.78, accessed on 4 November 2024), indicating that significant numbers of β-mannanases were identified via recent high-throughput sequencing efforts. Depending on the organism or substrate variation, the properties of mannanases vary greatly; generally, their pH ranges from 1 to 10, optimum temperature from 30 to 93 °C, km value from 0.6 to 2.9 mM (Mannan substrate), and pI value from 3.6 to 9 (BRENDA:EC3.2.1.78).

The crystal structures of GH26 enzymes adopt a (β/α)_8_ barrel structure, also known as the TIM barrel fold, a common structural motif in glycoside hydrolases. The active site is located within a cleft or groove on the enzyme surface, formed by loops connecting the β-strands and α-helices of the barrel [19]. The catalytic residues typically include a proton donor and a nucleophile/base, essential for the hydrolysis of glycosidic bonds; these residues are positioned in the active site, aligned for effective substrate binding and catalysis [20]. GH26 enzymes contain substrate-binding subsites that interact with the sugar backbone of mannans, ensure specificity, and stabilize the enzyme–substrate complex during catalysis [21]. Some GH26 mannanases display additional domains or loops that contribute to substrate specificity or enzyme stability. Comparative structural studies suggest that differences in loop regions around the active site can explain variations in substrate preferences among GH26 enzymes [22]. Recently, high-resolution images of GH26 mannanase crystal structures revealed key details about enzyme–substrate interactions, catalytic site architecture, and flexibility [23]; for example, structures bound with substrates or inhibitors demonstrate how mannose units are accommodated in the active site, providing insights into enzyme engineering for industrial purposes [24]. Additionally, crystallographic studies of GH26 members have been conducted for enzymes from various microbial sources, such as *Bacteroides*, *Cellvibrio*, and *Thermotoga* species, contributing to the understanding of thermostability, pH tolerance, and catalytic efficiency in these enzymes [23,25,26].

In this study, we searched for novel mannan-degrading enzymes using a metagenomic library constructed from the unculturable gut microorganisms of *H. illucens* larvae. Through functional screening, sequence-based annotation, and heterologous expression, we identified a novel glycoside hydrolase family 26 (GH26) mannanase gene with useful characteristics for industrial applications.

## 2. Results

### 2.1. Construction and Screening of a Metagenomic Library

The BSF can survive in highly contaminated habitats where noxious organic compounds, unfavorable pH, fluctuating temperature, and low dissolved oxygen are common. With their adaptability in mind, we expected that gut microorganisms might provide the BSF with effective hydrolyzing enzymes possessing unique characteristics. In order to investigate these enzymes, we previously constructed a BSF gut metagenomic library consisting of 92,000 individual fosmid clones, and we repeatedly screened them using different substrates [5,6].

We applied both function-based and sequence-driven screening to identify genes encoding hydrolyzing enzymes. Using function-based screening of the random shotgun fosmid library, we previously reported a novel cellulase gene from a positive fosmid clone, HC3, which uses carboxymethyl cellulase (CMC) as a substrate [5]. In this study, we further sequenced the full-length fosmid and searched for additional glycoside-hydrolyzing genes from the clone. The fosmid, HC3, harbored a 36,645 bp insert, and ORF analysis revealed a total of 25 codons; among them, we found six glycoside-hydrolyzing genes, including one putative α-galactosidase, three putative mannosidases, and two glucosidase-related proteins.

### 2.2. Sequence Analysis of a β-Mannanase Gene, ManEM6

**Figure 1:**
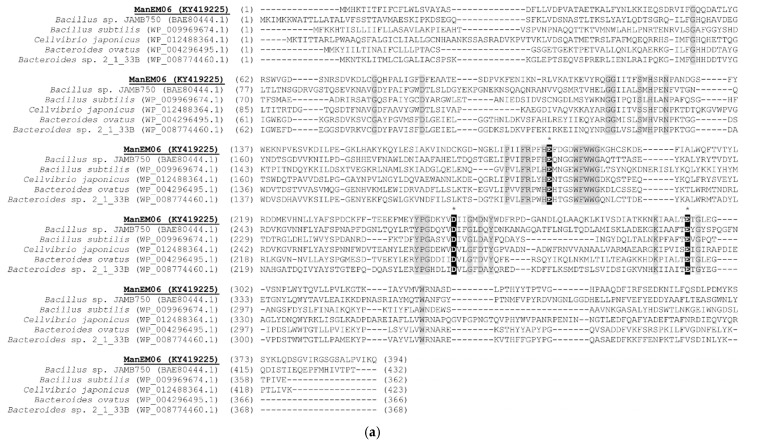

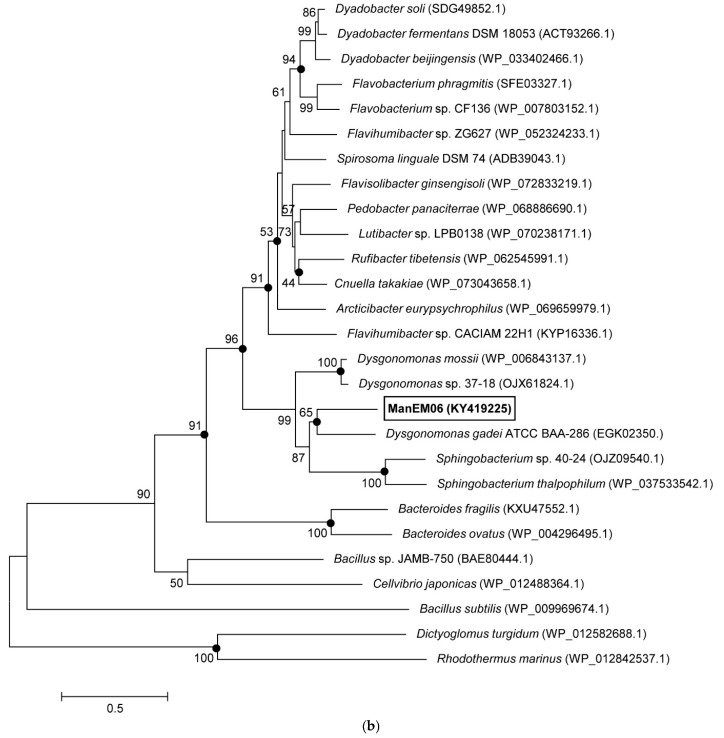
Multiple alignment and phylogenetic tree of ManEM6. (**a**) Multiple sequence alignment with glycoside hydrolase family 26 (GH26): β-endo-mannanase showed three putative catalytic residues (Glu189, Asp254, and Glu296), which are shown in dark-shaded letters. Asterisks (*) indicate consensus amino acids. Each protein sequence used for alignment was collected from GenBank, including *Bacillus* sp. JAMB750 [29], *Bacillus subtilis* [31], *Cellvibrio japonicas* [32], *Bacteroides ovatus* [33], *Bacteroides* sp. 2_1_33B [34]. (**b**) The phylogenetic tree of ManEM6 and other closely related enzymes was reconstructed using the neighbor-joining method (MEGA5.1 software). The protein sequences of related enzymes were retrieved from NCBI GenBank. Bootstrap values (>50%) at the nodes were based on 1000 replicates of the dataset. Solid circles indicate that the corresponding branches were also recovered in both the maximum parsimony and maximum likelihood trees. Bar, 0.2 substitutions per amino acid position.

The sequence length of the putative β-mannanase (denoted *ManEM6*) was 1185 bp, encoding a peptide of 394 amino acids (45.2 kDa and pI 5.46) (Supplementary File S1). A scan of the deduced amino acid sequence using the SignalP3.0 database predicted a microbial signal peptide sequence in the *N*-terminal 20 amino acids, with its putative cleavage site between Ala_20_ and Ser_21_. BlastP homology search of *ManEM6* showed the highest sequence identity (63%) with β-1,4-mannosidase from *Dysgonomonas mossii*. Further homologic comparison with the CAZy database (https://www.cazy.org/Glycoside-Hydrolases.html, accessed on 4 November 2024) revealed that *ManEM6* may belong to GH26 [27]. The presumed 374-amino-acid sequence without the *N*-terminal signal peptide sequence (fragment of 1125 bp, 42.8 kDa, and pI 5.29) also contained the conserved GH26 domain. Conserved domain analysis with other β-mannanases showed that the deduced protein of *ManEM6* contained three well-conserved motifs reported in the catalytic active site of GH26. The deduced protein of ManEM6 contained an acid/base-donor site (Glu_189_) [28], a catalytic nucleophile active site (Glu_296_) [29], and a putative catalytic residue (Asp_254_) [30], indicating inherent β-mannanase activity in ManEM6 (Figure 1a).

Phylogenetic analysis with other closely related β-mannanases classified ManEM6 into a separate clade, deep in the phylogenetic tree, demonstrating that it is a novel β-mannanase enzyme derived from an uncultured microorganism that is currently unknown (Figure 1b).

### 2.3. Heterologous Expression and Biochemical Characteristics of the Recombinant β-Mannanase

In order to investigate its functionality, *ManEM6* was expressed in *Escherichia coli*; however, protein induction of the whole *ManEM6* ORF (1182 bp without TAA termination codon) resulted in inclusion bodies, making the recombinant protein insoluble. Therefore, a trimmed recombinant *ManEM6* (denoted rManEM6), without the signal peptide sequence (1122 bp without the termination codon), was cloned, overexpressed in *E. coli* BL21 (DE3), and purified using metal-chelating chromatography under native conditions, as described above.

**Figure 2:**
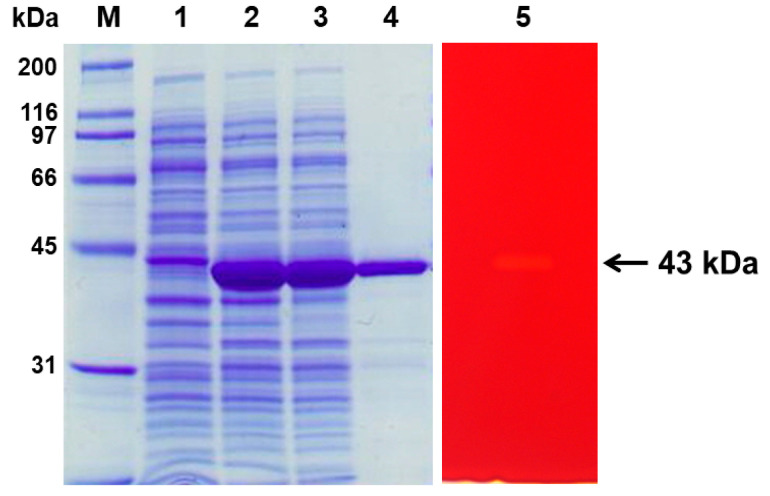
Overexpression and purification of rManEM6. ManEM6 without putative *N*-terminal signal peptide sequence was cloned into plasmid pET21a (+) expression vector and expressed in *E. coli* BL21(DE3). SDS–PAGE analysis shows a homogeneous 43 kDa protein. Lanes: M, molecular weight marker; Lane 1, total cellular protein from uninduced cells; Lane 2, induced total cellular protein; Lane 3, induced soluble fraction; Lane 4, purified rManEM6 protein; Lane 5, zymogram of rManEM6 on polyacrylamide gel with 0.2% LBG (locust bean gum) and stained with Congo-red.

**Figure 3:**
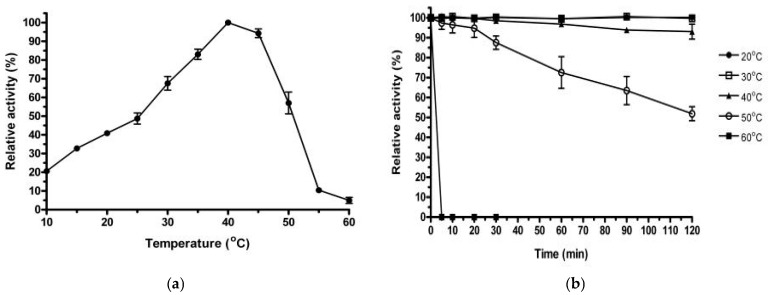

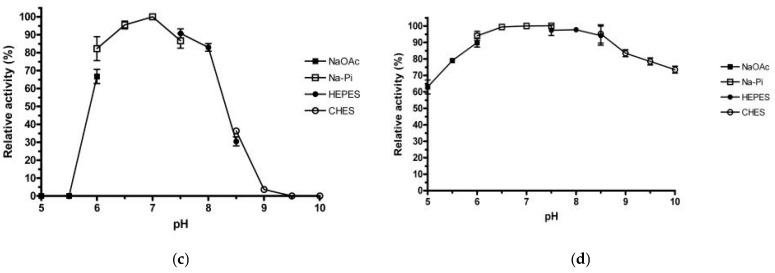
Biochemical characterization of rManEM6. (**a**) The enzyme activity of rManEM6 (0.2 μg) was measured at various temperatures in 50 mM sodium phosphate (pH 7.0) for 10 min, exhibiting >50% hydrolytic activity in the range of 25–50 °C with an optimum at 40 °C. (**b**) The thermostability of rManEM6 was examined at 40 °C for 10 min after 4 h pre-incubation at the given temperature. (**c**) pH inhibition was monitored in the following 50 mM buffers: sodium acetate buffer (NaOAc, pH 5.0–6.0), sodium phosphate buffer (Na-Pi, pH 6.0–7.5), 4-(2-hydroxyethyl)-1-piperazineethanesulfonic acid buffer (HEPES, pH 7.5–8.5), and 2-(cyclohexylamino) ethanesulfonic acid buffer (CHES, pH 8.5–10.0). (**d**) To verify pH stability, the enzyme was pre-incubated in 50 mM buffer with different pH values at 4 °C for 15 h, and residual activity was examined. Each β-mannanase assay was carried out by measuring the amount of mannose released from locust bean gum (0.5% [*w*/*v*]) under standard assay conditions, as described in the Materials and Methods section. Error bars represent SEM from triplicate results (*p* ≤ 0.01).

SDS–PAGE analysis showed a homogeneous band of 43 kDa, which was consistent with the estimated molecular weight (Figure 2). Zymographic staining of the purified rManEM6 on polyacrylamide gels revealed the active band of mannanase at a position corresponding to the molecular mass of the protein on SDS–PAGE. The enzymatic characteristics of rManEM6 were monitored using locust bean gum galactomannan, and it showed maximum hydrolytic activity at 40 °C and pH 7.0. Interestingly, rManEM6 showed relatively high activity and stability at low temperatures, retaining 70, 40, and 20% of its maximum activity at 30, 20, and 10 °C, respectively. rManEM6 stably maintained more than 90% activity below 40 °C, whereas it quickly lost its glycosyl hydrolase activity at temperatures higher than 50 °C. The stable half-life of rManEM6 was 2 h at 50 °C; it was most active in a pH range of 6.0 to 8.0. Meanwhile, pre-exposure of rManEM6 to acidic and alkaline conditions did not significantly affect its enzymatic activity; even after long-term pre-incubation in acidic or alkaline pH for 15 h at 4 °C, rManEM6 maintained stable activity over pH 5.0–10.0, with at least 70% residual activity retained (Figure 3).

To determine the effects of chemical reagents, rManEM6 was mixed with various organic solvents, detergents, metal ions, and denaturing reagents and was fairly stable toward many hydrophilic polar solvents (log*P*_ow_ ≤ 0.28), with more than 80% of its original activity maintained at 10% (*v*/*v*) in solvents such as dimethyl sulfoxide, dimethyl formamide, methanol, ethanol, and acetone; additionally, the enzyme remained up to 30% of its residual activity even in the presence of 10% hydrophobic nonpolar solvents, including n-pentanol, n-hexanol, benzene, toluene, cyclohexane, and n-hexane.

**Figure 4:**
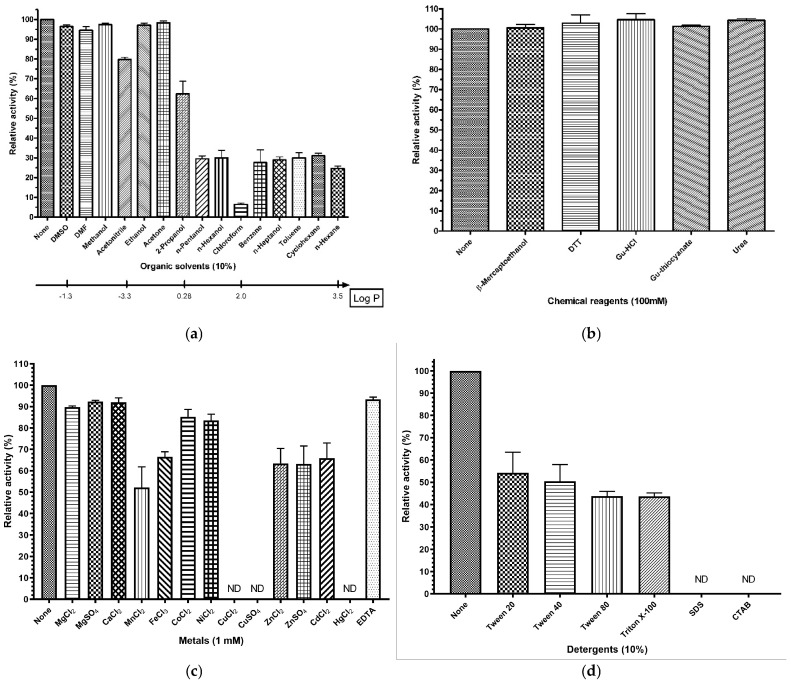
Enzymatic stability of rManEM6 against enzyme inhibitors. (**a**) The same amount of rManEM6 protein (0.2 μg) was pre-incubated at 30 °C for 30 min with different organic solvents (10% [*v*/*v*]) in 50 mM sodium phosphate buffer (pH 7.0, 200 μL), and residual β-mannanase activity was measured at 40 °C for 10 min with locust bean gum (0.5%, [*w*/*v*]) as a substrate. The bar represents the log-polarity of each organic solvent. DMSO, dimethyl sulfoxide; DMF, dimethylformamide. (**b**) To determine stability under chemical reagents, rManEM6 (0.2 μg protein) was pre-incubated with various denaturants or reducing agents at 30 °C for 30 min, and residual activity was measured. Gu-HCl, guanidine hydrochloride; gu-thiocyanate, guanidine thiocyanate. (**c**) An enzyme assay was performed after pre-incubation of rManEM6 with 1 mM of each of the indicated metal ions at 30 °C for 30 min. The relative amount of mannose produced was compared with that in the standard reaction. (**d**) To examine the effects of detergents on rManEM6 stability, the residual activity from pre-incubated enzyme (in 10% ([*v*/*v*] or [*v*/*w*]) detergent at 30 °C for 30 min) was measured. SDS, sodium dodecyl sulfate; CTAB, cetyltrimethylammonium bromide. The activity measured in the absence of any of the chemical compounds is shown as 100% (*p* ≤ 0.01). ND, not detectable.

We found that purified rManEM6 exhibited denaturation stability; even after exposure to high concentrations of denaturants and disulfide-reducing agents, it retained its original activity. Moreover, rManEM6 retained a significant amount of enzymatic activity, even under higher concentrations of urea, such as 2 M and 5 M, with residual activity of 55.9% and 15.7%, respectively; in contrast, several divalent metal ions affected rManEM6’s stability. Compared with divalent Mg^2+^, Ca^2+^, Co^2+^, or Ni^2+^, notable activity losses were observed with the addition of Mn^2+^, Fe^2+^, Cu^2+^, Zn^2+^, or Cd^2+^ ions, while divalent Cu^2+^ or Hg^2+^ ions completely abolished rManEM6 activity. Metal-chelating ethylenediaminetetraacetic acid (EDTA) did not inhibit rManEM6-mediated substrate hydrolysis, indicating that rManEM6 is a non-metalloprotein. In addition, both non-ionic and ionic detergents drastically inhibited rManEM6 activity; when incubated with non-ionic detergents (Tween 20, Tween 40, Tween 80, and Triton X-100), its activity decrease was substantial (~50% inhibition), while strong ionic surfactants, such as CTAB (cationic) and SDS (anionic), completely inhibited enzyme activity (Figure 4).

The degradation of glucomannans and galactomannans by mannan-degrading enzymes is greatly affected by the extent and pattern of branching sugar moieties in the mannan backbone. Among major mannan-degrading enzymes, both β-mannosidase and β-glucosidase act as exo-type enzymes, hydrolyzing β-1,4-linked mannosides (or β-1,4-d-glucopyranosides) from the non-reducing end of the glucomannans; furthermore, exo-type α-galactosidases cleave α-1,6-linked galactose, releasing branched sugar moieties from galactomannans [10].

The purified rManEM6 was able to hydrolyze locust bean gum galactomannan, Konjac glucomannan, β-1,4-mannan, and guar gum galactomannan, showing the highest specific activity against locust bean gum; meanwhile, rManEM6 could not hydrolyze natural polysaccharides such as gum arabic, pectin, xylan, pullulan, starch, and cellulose, in which non-mannoside sugar backbones are linked by either α-(1→4) or β-(1→4).

In addition, rManEM6 did not act on exo-type mannan residues in *p*-Nitrophenyl-β-mannopyranoside, indicating it is not a β-mannosidase; these results demonstrate that rManEM6 may be an endo-type β-mannanase (Table 1).

**Figure 5:**
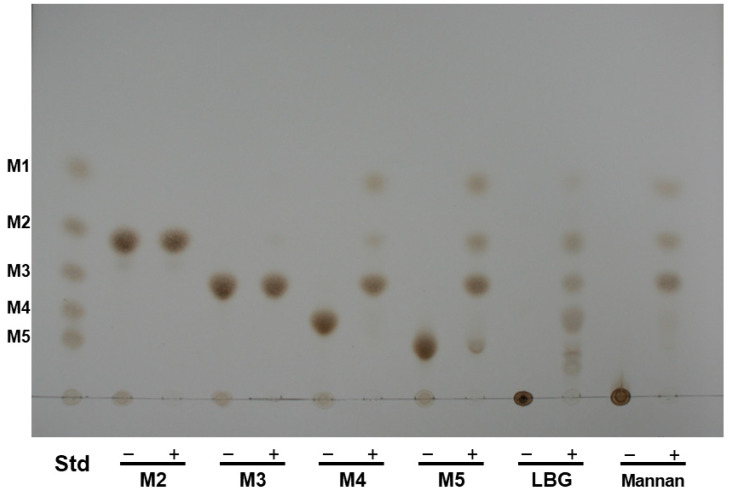
Thin layer chromatography analysis of hydrolysis products of mannose oligo- and polysaccharides by rManEM6. Enzymatic hydrolysis of each substrate (0.5%, *w*/*v*) was performed either with (+lanes) or without (–lanes) ManEM6 in 50 mM sodium phosphate buffer (pH 7.0) at 40 °C for 12 h. Lane Std, mannooligosaccharide standards: mannose (M1), mannobiose (M2), mannotriose (M3), mannotetraose (M4), and mannopentaose (M5).

The hydrolysis products from mannose oligo- and polysaccharides by rManEM6 were analyzed using TLC, and the enzyme randomly hydrolyzed only the internal bonds within the main backbone of mannose saccharides with a degree of polymerization ≥ 4, showing no activity on mannobiose and mannotriose (Figure 5).

## 3. Discussion

Mannanases degrading β-mannans (or heteromannans) are ubiquitous in nature and have been identified from bacteria, fungi, animals, and plants [35,36,37]; GH5 endo-β-mannanases are primarily produced by eukaryotes, such as fungi, plants, and animals [14]. Differences include that mannanases derived from lower animals are mainly found in *Aplysia Kurodai* [38], *Haliotis discus hannai* [39], and *Mytilus edulis* [40], which live in marine environments and may have enzyme properties that are adapted to withstand specific extreme environments and maintain activity at high temperatures or salinity. On the other hand, mannanases derived from the gut microbiomes of insect larvae are optimized for the digestive system of insects and may be more sensitive to temperature and pH changes, and their expression may be regulated by the composition of gut microbiota and dietary intake. Both types of mannanase are enzymes that decompose mannan, generally have similar three-dimensional structures, and play a role in hydrolyzing mannan, a component of plant cell walls, and converting it into monosaccharides. Both families share common roles in the digestion and metabolism of nutrients in these organisms. GH5 mannanases prefer or are optimized against glucomannan attacks in the cell walls of angiosperms [31]; on the contrary, GH26 endo-β-mannanases are found mainly from bacterial sources [41,42]. GH26 endo-β-mannanases showed strict conservation of acid/base and nucleophile residues (both are glutamic acids) [43], and although the bacterial origin of ManEM6 is not currently clear, we found that ManEM6 showed GH26 β-mannanase domains, along with well-conserved catalyst residues in the ORF, implying its bacterial origin from an insect intestinal microbiome.

For decades, functional metagenomic approaches were applied to discover novel genes from unculturable environmental microorganisms [44,45]. Based on metagenomic tools, previous studies have successfully isolated enzymes from environmental metagenomes that exhibit excellent properties suitable for industrial applications [46,47,48]; likewise, further surveys for novel β-mannanases in metagenomes provided enzymes with unique biochemical properties [49]. Despite the recent elucidation of the ORF sequences of many β-mannanases due to high-throughput sequencing results, we could not find homologous sequences for *ManEM6* from current metagenome and UniProt/Swiss-Prot databases in repetitive NCBI (http://www.ncbi.nlm.nih.gov/BLAST, accessed on 4 November 2024) BlastP searches.

Even with its enormous potential, current reports of GH26 β-mannanases from environmental resources are limited; among 1454 GH26 mannanase genes registered in the CAZy database, only 15 GH26 mannanase genes have known metagenome origins (http://www.cazy.org/, accessed on 4 November 2024). Moreover, few metagenome-derived GH26 β-mannanases are reported currently from insect intestines, except one GH26 endo-β-mannanase from the termite gut [50].

Endo-β-mannanases often display catalytic carbohydrate-binding module (CBM) domains, which confer carbohydrate-binding activity; their major roles include binding the enzyme to the substrate and enhancing its catalytic activity towards insoluble mannans [51]. However, the CBM association of recombinant mannanases has various effects on enzyme stability [52]. Previous studies identified various CBM domains from β-mannanases inside or flanking regions of the ORF, which include CBM 1~5, 10, 16, 23, 27, 32, 35, 59, and 72 (http://www.cazy.org/Carbohydrate-Binding-Modules.html, accessed on 4 November 2024) [53], but we found no known CBM domain sequences in the region of upstream and downstream sequences of ManEM6 ORF.

Despite the absence of CBM domains, ManEM6 showed clear substrate specificity for optimum catalytic activities. ManEM6 demonstrated higher galactomannanase activity toward the substrate locust bean gum than for guar gum, depending on the order of galactomannan substitution level. The locust bean gum galactomannan is primarily composed of one galactose per four mannose backbones, while the ratio becomes lower, to one galactose per two mannose backbones, in guar gum galactomannan [11]. ManEM6 could not hydrolyze glucose, galactose, galacturonic acid, xylose backbone, or exo-mannoside residues; these substrate specificity results suggest that ManEM6 acts specifically on endo-β-1,4-link of the galactomannan backbone, indicating that ManEM6 prefers longer endo-type mannose residues for proper enzyme catalysis.

Mannans are most common in plant seeds, fruits, and softwoods. Endo-β-mannanases are important to many industrial applications, such as food, feed, natural detergent, and bioethanol production, as well as in the oil drilling process, for efficient and cost-effective hydrolysis of polysaccharides [11,52,54,55]; in order to exploit them for industrial-scale applications, several endo-β-mannanases from bacteria have been cloned and expressed in *E. coli* [15,56,57].

In addition to unique amino acid sequences, ManEM6 exhibited another applicable feature. Increased activity and functionality of the enzyme under unusual conditions are crucial requisites for the bioconversion of hemicellulose in various industrial processes [15]. It was demonstrated that ManEM6 is active at low temperatures, showing over 20% of its maximum activity at 20 °C; to date, only a few mannanases have been reported to be low-temperature or cold-active, including five GH26 mannanases from *Sphingomonas* sp. JB13, *Sphingobacterium* sp. GN25, *Aspergillus niger* CBS513.88, *Bacillus licheniformis*, Verrucomicrobiae DG1235, a GH5 mannanase from *Cryptopygus antarcticus*, and a family-unidentified mannanase from *Falvobacterium* sp. Although the enzyme’s stability was lower than those of other thermostable enzymes at high temperatures [52], ManEM6 exhibited about 100% residual activity at room temperature and retained more than 70% of its control activity for 1 h at 50 °C; these enzymatic properties can be beneficial to the starch-processing and detergent industries, where they facilitate stable biocatalysts above room temperature and environmentally friendly effects. The recombinant ManEM6 was stable over a broad pH range, retaining more than 60% of its initial activity after 15 h incubation at pH 5~10. Moreover, ManEM6 is likely suitable for nonaqueous biocatalysis because it maintains a significant amount of enzymatic activity in selected organic solvents. The reported low-temperature- or cold-active mannanases were demonstrated to be salt-resistant or protease inhibitor-resistant, but no organic solvent resistance has been reported so far. Additionally, Man EM6 was highly stable against many different chemical reagents, including β-mercaptoethanol, DTT, urea, and guanidium salts.

The low-temperature activity and organic solvent resistance of ManEM6 remaining stable over a broad pH range is of great importance, especially in industrial processes where harsh conditions are employed; in addition, organic solvent stability provides important economic advantages in terms of less byproduct, higher productivity, and lower processing cost [58].

Currently, the highest specific activity obtained after the cloning and expression of endo-β-mannanase in *E. coli* BL21 (DE3) was 8406 U/mg through high cell density fermentation [55,59]. Compared to the highest specific activity, we assume less specific activity may be due to the absence of a CBM module, as we could not find any available modules in the ManEM6 ORF; therefore, to facilitate the association between enzyme and substrate, protein engineering, by attaching a CBM to ManEM6, may increase specific activity toward insoluble mannans.

In summary, the present work reports the molecular cloning, heterologous expression, and biochemical characterization of a novel endo-β-mannanase, ManEM6, from the insect larvae gut metagenome. ManEM6 exhibited its most efficient hydrolysis toward locust beam galactomannan, optimum at pH 7.0 and 40 °C. Although the underlying mechanism remains to be determined, the activity of ManEM6 is stable against various chemical denaturants and most polar organic solvents; its durability, along with its low-temperature-active and alkali-stable nature, places ManEM6 as an attractive supplement of biocatalysts in commercial softwood bioconversion processes that require heavy burdens of noxious components.

## 4. Materials and Methods

### 4.1. Cloning of β-Mannanase from Larvae Gut Metagenome

Construction of a metagenomic fosmid library from the gut of *Hermetia illucens* was described previously [5,6]; screening and selection of glycoside-hydrolyzing clones from the fosmid library on carboxymethyl cellulose (Sigma, Anseong, Republic of Korea) were conducted as previously described [5]. Fosmids showing substrate hydrolysis were subjected to full-length sequencing and searched for glycoside-hydrolyzing genes. A sequencing reaction using Big Dye Kit (Applied Biosystems, Seoul, Republic of Korea) was outsourced to Solgent Co., Seoul, Republic of Korea. BLAST searching was performed on the National Center for Biotechnology Information database (NCBI, http://www.ncbi.nlm.nih.gov/, accessed on 4 November 2024). Open reading frames (ORFs) were estimated using VectorNTI ver. 11 (Invitrogen, Waltham, MA, USA). One ORF, encoding a β-mannanase (denoted ManEM6), was identified with sequence comparison; the nucleotide sequence of the ManEM6 gene was deposited into the NCBI database under GenBank accession number KY419225. Multiple sequence alignments and phylogenetic tree construction for the ManEM6 gene were carried out using ClustalW (ver. 1.2.2) and MEGA software (ver. 5.1), respectively. To predict putative signal peptides, the SignalP 3.0 server (http://www.cbs.dtu.dk/services/SignalP/, accessed on 4 November 2024) was used. The routine cloning of the DNA fragments into various vectors was achieved in *Escherichia coli* DH5α.

### 4.2. Heterologous Expression and Purification of the Recombinant Protein

The putative β-mannanase gene, ManEM6, was PCR-amplified from a fosmid clone, HC3, using the following forward and reverse primers: 5′-ATGGGTCGCGGATCCTCCGACTTTCTCTTAG-3′ and 5′-GTGGTGGTGCTCGAG-TTATTGCTTGATGACG-3′ (*Bam*HI and *Xho*I restriction enzyme sites are underlined) and cloned into a *Bam*HI-*Xho*I pre-digested pET21a (+) vector (Novagen, Madison, WI, USA) using an In-Fusion HD cloning kit (Clontech, Mountain View, CA, USA). The construct was then transformed into *E. coli* BL21 (DE3) for heterologous protein expression. The optimum induction condition was found to be 20 °C for 20 h under 0.5 mM IPTG. After protein induction, transformants were harvested and disrupted, as described previously [6]. For recombinant protein purification, cell debris was removed via centrifugation at 13,000× *g* for 30 min at 4 °C. The soluble supernatant was loaded onto pre-equilibrated TALON metal affinity resin (BD Biosciences, Clontech, USA), and the recombinant protein with *C*-terminal His-tag was purified as described previously [6]. The purity of the purified protein was determined using 10% sodium dodecyl sulfate-polyacrylamide gel electrophoresis (SDS-PAGE). The protein concentration was determined with a Bradford assay kit (Bio-Rad, Hercules, CA, USA) following the manufacturer’s recommendation. The zymogram analysis of the purified enzyme was conducted using a previously described method. After 10% SDS–PAGE with 0.2% locust bean gum (LBG) as the substrate, SDS-PAGE gel was soaked in 50 mM sodium phosphate buffer, pH 7.0 containing 2.5% Triton X-100 for 30 min, to remove SDS, and then incubated in the same buffer without Triton X-100 for 1 h to allow the protein into the gel, which was then stained with Congo-red (1%) for 30 min and destained with 1 M NaCl until a clear zone was observed against the red background.

### 4.3. Biochemical Characterization of the Purified Recombinant Protein

All chemical reagents and substrates were obtained from Sigma-Aldrich (Seoul, Korea) unless stated otherwise. β-mannanase activity was evaluated using either the *p*-Nitrophenyl mannopyranoside method or the 3,5-dinitrosalicylic acid (DNS) detection method, depending on the substrate used. The standard activity assay was conducted by mixing diluted rManEM6 enzyme (0.2 µg protein) and the substrates (LBG (0.5%, [*w*/*v*]) or 0.1 mM *p*-nitrophenyl-β-pyranosides) in 50 mM sodium phosphate buffer (pH 7.0, 200 μL) at 40 °C for 10 min, and then, the amount of mannose or *p*-Nitrophenol released was determined spectrophotometrically at 540 nm or 405 nm, respectively. The optimum temperature and pH were measured via assay for 10 min at different temperatures (10–60 °C with an interval of 5 °C) and pH values (5.0–10.0 with an interval of 0.5), using locust bean gum as a substrate. Thermostability was monitored after pre-incubating the enzyme in the absence of substrate for 4 h at different temperatures, ranging from 20 to 60 °C; then, residual activity was measured using an equal amount of enzyme taken at regular intervals. Similarly, to monitor pH stability, the pre-incubated enzyme was prepared by placing the enzyme in different pH buffers without any substrate for 15 h at 4 °C. pH stability was compared with residual activity measured under the same standard assay conditions. The substrate specificity of the purified protein was investigated by measuring the hydrolytic activities of the enzyme toward various substrates under standard assay conditions, replacing LBG with the respective substrates, including LBG, konjac glucomannan, β-1,4-mannan, guar gum galactomannan, gum arabic, pectin from citrus fruits, birchwood xylan, oat spelt xylan, pullulan, soluble starch, carboxymethylcellulose, *p*-Nitrophenyl-β-mannopyranoside, *p*-Nitrophenyl-β-glucopyranoside, and *p*-Nitrophenyl-β-cellobioside. Enzymatic stability in the presence of inhibitors such as organic solvents, protein-denaturing reagents, detergents, divalent metal ions, and detergents was also examined. For experimental controls, the enzyme was pre-incubated without substrate under given additives at 30 °C for 30 min before measuring the residual activity. In activity tests, each inhibitor was added separately into the reaction mixture to investigate their effects on rManEM6. In all of the tests, the residual activity of enzyme pre-incubated in the absence of any additives was defined as 100%, and the relative amount of mannose produced in a standard assay was compared. The substrate preference of rManEM6 toward various substrates was determined by measuring hydrolytic activity under standard assay conditions. The amount of mannose released from macromolecular polysaccharides (0.5%, [*v*/*v*]) by rManEM6 was determined according to DNS colorimetric methods at 540 nm. All biochemical experiments were performed in triplicate.

### 4.4. Thin-Layer Chromatography (TLC) Analysis of Hydrolyzed Products

The hydrolysis reactions of mannanase were conducted by incubating an appropriate amount of the purified protein with 0.5% (*w*/*v*) substrate in 50 mM sodium phosphate buffer (pH 7.0) at 40 °C for 2 h. The aliquots of the reaction products were spotted on Silica Gel 60 TLC plates (Merck, Darmstadt, Germany), using a solvent of n-butanol/acetic acid/water (2:1:1 (*v*/*v*/*v*)). After development, the hydrolysis products were visualized by spraying the gel plate with freshly prepared 10% (*v*/*v*) H_2_SO_4_ in ethanol and heating at 120 °C for 10 min. Manno-oligosaccharides (mannose (M1), mannobiose (M2), mannotriose (M3), mannotetraose (M4), and mannopentaose (M5); Megazyme, Bray, Ireland) were used as standards.

## 5. Conclusions

A mannan-degrading gene, ManEM6, encoding a novel hydrolytic enzyme, was discovered from a fosmid library of an unculturable intestinal microorganism, *Hermetia illucens*. The enzyme encoded by this gene had the highest identity (78%) with the endo-1,4-β-mannosidase of *Dysgonomonas mossii*, and the recombinant protein rManEM6 showed the highest activity at 40 °C and pH 7.0. rManEM6 used β-1,4-glycosidic mannoses as substrates and showed the highest enzymatic activity toward locust bean gum galactomannan; it was also demonstrated to have endo-form mannosidase activity through its inability to degrade *p*-nitrophenyl-β-pyranosides. rManEM6 was highly stable under stringent conditions, including those of polar organic solvents, as well as reducing and denaturing reagents. In conclusion, ManEM6 is an attractive candidate for the degradation of mannan under high-organic-solvent and protein-denaturing processes in the food and feed industries.

## Figures and Tables

**Table 1:** Substrate preferences of the purified ManEM6.

Substrate	Specific Activity ^a^ (U/mg)
Locust bean gum galactomannan	508.77 ± 4.57
Konjac glucomannan	374.15 ± 12.69
β-1,4-mannan	279.92 ± 20.67
Guar gum galactomannan	39.02 ± 3.71
Gum arabic	ND ^b^
Pectin from Citrus fruits	ND
Birchwood xylan	ND
Oat spelt xylan	ND
Pullulan	ND
Soluble starch	ND
Carboxymethylcellulose	ND
*p*-Nitrophenyl-β-mannopyranoside	ND
*p*-Nitrophenyl-β-glucopyranoside	ND
*p*-Nitrophenyl-β-cellobioside	ND

^a^ Substrate specificity was measured under standard assay reaction conditions, as described in the Materials and Methods section. One unit was defined as the amount of enzyme required to make 1 μmol of product (mannose or *p*-Nitrophenol) per minute. Standard errors of mean from biological triplicates are shown. ^b^ ND, not detectable.

## Data Availability

Data are contained within the article and Supplementary Materials.

## Supplementary Materials

The following supporting information can be downloaded at https://www.mdpi.com/article/10.3390/ijms26010216/s1.
