# Supplementary material for: Isolation of a Novel Low-Temperature-Active and Organic-Solvent-Stable Mannanase from the Intestinal Metagenome of Hermetia illucens
Source: Int J Mol Sci. 2024 Dec 30;26(1):216. doi: 10.3390/ijms26010216 (PMC11720594; doi:10.3390/ijms26010216)
Supplement: Supplementary file 1 [file ijms-26-00216-s001.zip › Supplementary File S1. ManEM6 Nucleotide and Amino Acid Sequences.pdf]

>ManEM06_KY419225_1185bp

ATGATGCACAAAACAATCACTTTTATTTTTTGCTTCTTGTGGCTCTCTGTTGCATATGCTTCCGACTTTCTCTTAG
TCGACCCAGTGGCGACAGCCGAGACCAAAGCCTTGTTTTATAATCTGAAAAAGATAGAACAATCGGATCGGG
TTATTTTCGGACAACAGGACGCTACCTTATACGGTCGATCGTGGGTAGGCGACAGTAACAGAAGCGATGTGAA
AGACCTCTGTGGGCAACATCCGGCATTGATTGGTTTTGATTTTGAAGCTGCTACAGAAAGTGATCCTGTAAAAT
TCGAAAACATAAAGAACCGGTTGGTCAAAGCAACTAAAGAAGTTTACCGCCAAGGTGGAATTATAACCTTTTC
GTGGCATAGCAGAAACCCTGCTAATGATGGAAGTTTCTATTGGGAGAAGAATCCTGTGGAATCGGTAAAAGAT
ATTCTTCCCGAAGGAAAGCTTCACGCGAAATATAAACAATATCTGGAATCTATAGCCAAAGTCATAAATGATTGT
AAAGGAGATAATGGCGAACTTATTCCTATTATTTTTCGTCCTTTCCATGAATTCGACGGCGATTGGTTCTGGTGG
GGAAAGGGACATTGTTCGAAAGATGAATTTATTGCACTTTGGCAATTTACCGTTACCTATCTCAGGGATGATAT
GGAGGTGCATAATCTTCTTTATGCTTTCTCTCCTGATTGTAAATTCTTTACTGAAGAAGAATTTATGGAGTATTAT
CCGGGAGATAAATATGTGGATATTATCGGAATGGACAATTATTGGGACTTTAGACCTGATGGTGCAAATGATCTT
CAACTCGCTGCACAAAAATTAAAGATCGTTTCGGATATTGCCACTAAAAAGAATAAGATTGCAGCTTTAACCG
AAACAGGATTAGAGGGGGTAAGCAATCCGCTTTGGTACACCCAGGTGTTGCTACCCGTTTTAAAGGGTACAA
AGATTGCGTATGTAATGGTATGGCGAAATGCGAGCGATTTACCAACGCATTATTATACACCGACAGTGGGACAT
CCTGCGGCTCAGGATTTCATCCGTTTTTCGGAAGATAAGAATATTTTATTCCAATCGGATTTACCTGATATGTACA
AAAGCAGTTATAAGTTACAAGACAGTGGGGTAATTAGAGGCAGTGGCTCTGCCCTGCCCGTCATCAAGCAATAA

>ManEM06_KY419225_394 Amino Acids

MMHKTITFIFCFLWLSVAYASDFLLVDPVATAETKALFYNLKKIEQSDRVIFGQQDATLYGRSWVGDSNRSDVKDL
CGQHPALIGFDFEAATESDPVKFENIKNRLVKATKEVYRQGGIITFSWHSRNPANDGSFYWEKNPVESVKDILPEG
KLHAKYKQYLESIAKVINDCKGDNGELIPIIFRPFHEFDGDWFWWGKGHCSKDEFIALWQFTVTYLRDDMEVHN
LLYAFSPDCKFFTEEEFMEYYPGDKYVDIIGMDNYWDFRPDGANDLQLAAQKLKIVSDIATKKNKIAALTETGLEG
VSNPLWYTQVLLPVLKGTKIAYVMVWRNASDLPTHYYTPTVGHPAAQDFIRFSEDKNILFQSDLPDMYKSSYKL
QDSGVIRGSGSALPVIKQ
